# Validation of a single biopsy approach and bolus protein feeding to determine myofibrillar protein synthesis in stable isotope tracer studies in humans

**DOI:** 10.1186/1743-7075-8-15

**Published:** 2011-03-09

**Authors:** Nicholas A Burd, Daniel WD West, Tracy Rerecich, Todd Prior, Steven K Baker, Stuart M Phillips

**Affiliations:** 1 Exercise Metabolism Research Group, Department of Kinesiology, McMaster University, Hamilton, Ontario, Canada; 2 Michael G. DeGroote School of Medicine, Department of Neurology, McMaster University, Hamilton, Ontario, Canada

## Abstract

**Background:**

Minimizing the number of muscle biopsies has important methodological implications and minimizes subject discomfort during a stable isotope amino acid infusion. We aimed to determine the reliability of obtaining a single muscle biopsy for the calculation of muscle protein fractional synthetic rate (FSR) as well as the amount of incorporation time necessary to obtain that biopsy after initiating a stable isotope infusion (Study 1). The calculation of muscle protein FSR requires tracer steady-state during the stable isotope infusion. Therefore, a second aim was to examine if steady-state conditions are compromised in the precursor pools (plasma free or muscle intracellular [IC]) after ingestion of a tracer enriched protein drink and after resistance exercise (Study 2).

**Methods:**

Sixteen men (23 ± 3 years; BMI = 23.8 ± 2.2 kg/m^2^, means ± SD) were randomized to perform Study 1 or Study 2 (n = 8, per study). Subjects received a primed, constant infusion of L-[*ring*-^13^C_6_]phenylalanine coupled with muscle biopsies of the vastus lateralis to measure rates of myofibrillar protein synthesis (MPS). Subjects in Study 2 were fed 25 g of whey protein immediately after an acute bout of unilateral resistance exercise.

**Results:**

There was no difference (P = 0.3) in rates of MPS determined using the steady-state precursor-product equation and determination of tracer incorporation between sequential biopsies 150 min apart or using plasma protein as the baseline enrichment, provided the infusion length was sufficient (230 ± 0.3 min). We also found that adding a modest amount of tracer (4% enriched), calculated based on the measured phenylalanine content of the protein (3.5%) in the drink, did not compromise steady-state conditions (slope of the enrichment curve not different from zero) in the plasma free or, more importantly, the IC pool (both P > 0.05).

**Conclusions:**

These data demonstrate that the single biopsy approach yields comparable rates of muscle protein synthesis, provided a longer incorporation time is utilized, to that seen with a traditional two biopsy approach. In addition, we demonstrate that enriching protein-containing drinks with tracer does not disturb isotopic steady-state and thus both are reliable techniques to determine rates of MPS in humans.

## Introduction

A common approach to quantify rates of skeletal muscle protein synthesis is to administer a primed continuous intravenous infusion of an isotopically labelled tracer amino acid with sequential muscle biopsies to determine the tracer incorporated into the product (e.g., muscle protein) over time [1]. The increment in tracer enrichment is divided by the precursor pool tracer enrichment from: plasma free amino acid, the tissue intracellular free amino acid (IC), or aminoacyl-tRNA (if obtainable) pool, to determine the fraction of the muscle protein pool that has been synthesized per hour [2]. However, under certain circumstances (e.g. methodological and/or ethical) it may be necessary to try to avoid this 'classic' sequential biopsy approach and instead determine muscle protein FSR from a single biopsy.

Smith and colleagues [3] have recently reported on the validity of an approach that used one muscle biopsy instead of the traditional sequential biopsy technique to determine the fractional synthetic rate (FSR) of muscle protein in human volunteers. These authors concluded that a single biopsy led to unreliable rates of muscle protein synthesis [3]. This conclusion was arrived at by assuming zero background as the initial enrichment and infusing a deuterated tracer. Previous investigations have infused carbon-labelled tracers and gas chromatography-combustion-isotope ratio mass spectrometry (GC-C-IRMS) as the primary analytical method to determine rates of muscle protein synthesis using the single biopsy approach, and the rates reported were physiologically interpretable [4-8]. An important distinction between these investigations [4-8] and that of Smith et al. [3] is that former studies utilized the initial enrichment from a body protein as a baseline measure, such as proteins isolated from plasma [4-6,8] or skin [7], and these investigations did not assume zero background for the initial enrichment. As a matter of fact, it has been determined that plasma protein enrichment more accurately reflects the pre-infusion enrichment of muscle protein [9]. However, this approach does necessitate that subjects have not received ^13^C tracer infusion as it relies on uniform labelling of amino acids incorporated into muscle protein [9]. Admittedly, the reliability of calculating rates of muscle protein synthesis using ^13^C background enrichment of a mixed plasma protein pellet coupled with a single biopsy obtained some time later has never been directly tested against the standard sequential biopsy technique. Moreover, it is unknown if the amount of tracer incorporation time allotted (i.e. short vs. long incorporation time) before obtaining the single biopsy will influence muscle protein FSR.

Our laboratory [4,6,8,10] and others [11-17] have a long standing interest in determining the FSR of muscle proteins after different acute exercise and feeding interventions. Therefore, a secondary aim of the investigation was to study the effects of ingesting a tracer enriched protein drink immediately after exercise on steady-state enrichments in the precursor pools (plasma free and IC pools). Specifically, steady-state precursor-product calculations can be used so long as a steady state enrichment of the amino acid tracer occurs in the precursor pools during the experiment. However, feeding protein would perturb steady state due to the introduction of exogenous unlabelled trace amino acids during the infusion. To reduce this possibility with a bolus protein feeding, we have added crystalline tracer to the protein drink (matched to the tracee amino acid concentration in the ingested protein at a level consistent with the predictable plasma free and IC pool enrichments) to minimize dilution of the tracer in the precursor pools and maintain steady-state enrichment [4,6,8,18]. However, the validity of such an approach, in maintaining precursor tracer steady-state enrichment, has recently been brought into question [19].

Indeed, it has been demonstrated that intact protein and free amino acids (i.e., tracer) have differential intestinal absorption kinetics such that the tracer will appear in the blood before the amino acids derived from the intact protein [20]. Further, if large quantities of crystalline tracer, greater than the abundance of the tracee in the ingested protein, are ingested measurements of whole body proteolysis and synthesis are falsely changed [20]; however, these results are only for whole-body kinetics [20]. This finding [20] has been suggested to invalidate the utilization of steady-state equations to calculate muscle protein FSR and ultimately yield flawed values [19]; however, this supposition has never been directly tested with a measurement of muscle protein FSR.

The purpose of this investigation was to test the reliability of commonly used, but recently questioned, approaches in tracer research for the determination of muscle protein FSR. Our first objective was to test the reliability of determining muscle protein FSR from mixed plasma protein and a single muscle biopsy. We also wished to determine if there is an optimal amount of incorporation time that should elapse before obtaining the single biopsy to reliably calculate muscle protein FSR. Our secondary objective was to improve the temporal resolution of the appearance of the tracer into systemic circulation after ingestion of a protein drink, with the tracer added as an appropriate fraction of the exogenously ingested protein, to examine if steady-state conditions are compromised, both in the plasma free and IC pools, after an acute bout of resistance exercise.

## Methods

### Subjects

Sixteen recreationally active males volunteered to participate in the study. All participants were deemed healthy based on their responses to a routine medical screening questionnaire.

### Ethics statement

All subjects were informed of the purpose of the study, the experimental procedures involved and all the potential risks involved before obtaining written consent. This study was approved by the local Health Sciences Research Ethics Board of McMaster University and conformed to standards for the use of human subjects in research as outlined in the fifth Declaration of Helsinki and with current Canadian Tri-council government funding agency guidelines for use of human subjects in research [21].

### Experimental design

**Figure 1:**
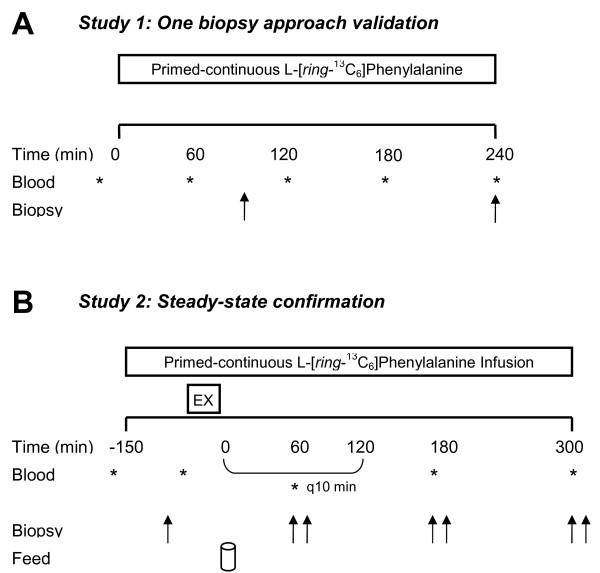
**Study protocol utilized for study 1 for the one biopsy approach validation**. (A) and study 2 for steady-state confirmation (B). Double arrows indicate bilateral biopsies were obtained at corresponding time points. During study 2, blood samples were obtained every 60 min; however, blood sampling was increased to every 10 min for 120 min after protein drink consumption and return to 60 min blood sampling thereafter.

Subjects were randomized to perform study 1 (the one biopsy approach confirmation experiment) or study 2 (i.e., the steady-state confirmation experiment) such that n = 8 was utilized for each study (Figure 1). Subjects were asked to adhere to their regular diets and refrain from physical activity for three days prior to the infusion protocols.

### Study 1: One biopsy approach validation experiment

Subjects (n = 8; 21 ± 4 years; BMI = 24.2 ± 2.8 kg/m^2^, means ± SD) reported to the laboratory at 0700 in the post-absorptive state and a catheter was inserted into a vein in the hand. A baseline blood sample was drawn before a 0.9% saline drip was started to keep the catheter patent for repeated blood sampling. A second catheter was inserted in the opposing arm for the primed-constant infusion of L-[*ring*-^13^C_6_]phenylalanine (prime: 2 μmol∙ kg^-1^; 0.05 μmol∙ kg^-1 ^∙min^-1^; Cambridge Isotope Laboratories Inc., Andover, MA, USA). The utilization of a single tracer to assess the reliability of the single biopsy approach allowed us to eliminate any potential variability shown to exist when making comparison between different tracers [22]. Muscle biopsies were obtained 90 min and 240 min after the start of the infusion. Myofibrillar protein synthesis rates were calculated from the biopsies obtained at 90 min and 240 min (2BX). The muscle biopsy obtained at 90 min and the mixed plasma protein fraction was used to assess the reliability of calculating rates of myofibrillar protein synthesis against the 2BX approach when utilizing a short incorporation time interval (1BX SHORT), whereas, the muscle biopsy obtained at 240 min was used to determine the reliability of a long incorporation time interval (1BX LONG). Blood samples were drawn every hour (Figure 1a). All experimental procedures for this study were performed within the same group of subjects.

### Study 2: Steady-state confirmation experiment

Subjects (n = 8; 22 ± 3 years; BMI = 23.2 ± 2.0 kg/m^2^, means ± SD) reported to the laboratory at 0700 in the post-absorptive state and the infusion was initiated according to study 1. A steady-state blood was obtained (Figure 1b) and subsequently subjects performed an acute bout of unilateral resistance exercise in which one leg performed a bout that consisted of 3 sets of 10-12 repetitions of leg press (MAX-1500 by Maxam, Hamilton, ON, Canada) and knee extension machine (Badger 2001 series by Magnum Fitness Systems, South Milwaukee, WI, USA) at their previously established 12 repetition maximum with 2 min rest between sets (EX-FED). The contralateral leg served as a non-exercised (i.e., resting) control (FED). After completion of the exercise bout, subjects consumed a drink containing 25 g of whey protein isolate (table 1). To minimize disturbances in isotopic equilibrium, the drinks were enriched to 4% with tracer according to a measured phenylalanine content of 3.56% in the whey protein (i.e., 0.04 × [0.0356 × 25 g] = 35.6 mg). Based on previous observations from our laboratory demonstrating that enriching protein drinks to 6 - 8% with crystalline tracer can cause a transient spike in blood enrichment, which did not disrupt steady-state conditions in the intracellular pool, immediately after drink consumption [4,6,18,23,24]. We instead, in this study, chose to use a lower tracer enrichment at a level of 4% tracer drink enrichment based on some pilot work (data not shown) showing minimal perturbation of blood and intracellular pools with lower tracer enrichments. Bilateral muscle biopsies were obtained at 60, 180, and 300 min post-exercise recovery. All muscle biopsies were obtained from the *vastus lateralis *and performed with a Bergström needle with procedures previously described [4,5,18]. Arterialized blood samples were drawn from a hand vein, which was warmed in a box heated to 60°C every 1 h in the fasted-state and every 10 min in the fed-states (Figure 1b) and processed as previously described [10]. All experimental procedures for this study were performed within the same group of subjects.

**Table 1:** Essential amino acid content of protein drinks in study 2

Essential Amino Acids	g/100 g
Isoleucine	5.4
Leucine	12.0
Lysine	10.8
Methionine	2.3
Phenylalanine	3.5
Threonine	4.4
Tryptophan	2.7
Valine	5.5

The numbers represent quantities in per 100 g (biPro, Davisco Foods, Le Sueur, MN, USA).

### Plasma analyses (Study 1 and 2)

Mixed plasma proteins were extracted from 200 μl of plasma by adding 500 μl of acetonitrile. The samples were spun at 10000 × *g *for 5 min at 4°C and the resultant supernatants were removed. The pellets were washed with 500 μl of distilled water and spun at 10000 × *g *for 5 min at 4°C. Supernatants removed and the protein pellets were washed twice more with 70% ethanol and lyophilized to dryness. Amino acids were liberated from the mixed plasma proteins by adding 1.5 ml of 6 M HCL and heated at 110°C for 24 h. Plasma [*ring*-^13^C_6_] phenylalanine enrichments were determined as previously described [25]. Blood amino acid concentrations were analysed by HPLC as previously described [26].

### Muscle analyses

Myofibrillar enriched proteins were isolated as previously described [5]. Amino acids were liberated by adding 1.5 ml of 6 м HCl and heating to 110°C for 24 h. Free amino acids were purified using cation exchange chromatography (Dowex 50WX8-200 resin; Sigma-Aldrich Ltd) and converted to their N-acetyl-n-propyl ester derivatives for analysis by GC-C-IRMS (Hewlett Packard 6890; IRMS model Delta Plus XP, Thermo Finnigan, Waltham, MA, USA). Derivatized amino acids were separated on a 30 m × 0.25 mm × 0.25 μm DB-5 column (temperature programme: 110°C for 2 min; 10°C∙min-^1 ^ramp to 240°C; 60°C∙min-^1 ^ramp to 300°C; hold for 5 min) prior to combustion. The isotopic abundances were expressed as the delta notation, δ^13^C per mil (‰) deviation from Pee Dee Belemnite (PBD) standard. Each sample was measured at least three times with a coefficient of variation always less than 2%.

Intracellular amino acids (IC) were extracted from a separate piece of wet muscle (~20 mg) with ice-cold 0.6 м perchloric acid. Muscle was homogenized on ice with a Teflon-coated pestle and then centrifuged at 10000 × *g *for 10 min at 4°C. The supernatant was then collected and this process was repeated two more times. All three supernatants were combined and taken as the IC and purified by cation-exchange chromatography and converted to their heptafluorobutyric (HFB) derivatives before analysis by GC-MS (models 6890 GC and 5973 MS; Hewlett-Packard, Palo Alto, CA, USA). Intracellular phenylalanine enrichment was determined using electron-impact ionization by monitoring ions 316 and 322 (*m *+0 and *m *+6, respectively).

### Calculation

The fractional synthetic rates (FSR) of myofibrillar proteins were calculated using the standard precursor-product method (Study 1):

For short tracer incorporation (1BX SHORT) determination, E_p2 _and E_p1 _are the protein bound enrichments from a muscle biopsy at 90 min and plasma proteins, respectively (see Figure 1A). For long tracer incorporation (1BX LONG), E_p2 _and E_p1 _are the protein bound enrichments from a muscle biopsy at 240 min and plasma proteins (see Figure 1A). For the two biopsies (2BX), E_p2 _and E_p1 _are the protein bound enrichments from a muscle biopsy at 240 min and a muscle biopsy at 90 min (see Figure 1A). The difference represents the change in bound protein enrichment between two time points; E_ic _is the mean intracellular phenylalanine enrichment from the biopsies; and *t *is the tracer incorporation time.

The δ per mil value (^13^C‰) was calculated using the (^13^C/^12^C) ratio of samples and the standard (Std) CO_2 _reference gas in the equation:

Where, the standard is referenced to the international PDB standard.

### Statistics

Differences in myofibrillar protein synthesis were tested by a one-factor (condition) analysis of variance (ANOVA) with repeated measures. Plasma free enrichments and blood amino acid concentrations were analyzed using one-factor (time) repeated measures ANOVA. Linear regression analyses were performed to assess the existence of a linear fit between variables. Pearson's *r *product moment correlation was used to examine the relationship between different variables. Tukey's post hoc test was performed to determine differences between means for all significant main effects and interactions. For all analyses, differences were considered significant at P < 0.05. All results are presented as means ± standard deviation (SD).

## Results

### Study 1: Single biopsy approach validation

#### Plasma and muscle intracellular free phenylalanine enrichment

Linear regression analysis revealed that the slopes of the plasma free enrichments over time curve (i.e. between 60 - 240 min) were not significantly different from zero (P = 0.30). Further, muscle IC enrichments were not significantly different (P = 0.60) from each other at biopsy time 1 (60 min: 0.037 ± 0.01 tracer·tracee^-1^) and time 2 (240 min: 0.040 ± 0.01 tracer·tracee^-1^). Thus, appropriate conditions were met for the application of the steady-state precursor product equation. Coefficients of variation in tracer enrichment over the duration of the infusion in the plasma free and IC pools after drink consumption were 12 ± 2.8 and 6 ± 2.8%, respectively.

#### Myofibrillar protein bound enrichments

The ^13^C phenylalanine enrichments expressed as the difference (δ) from the PDB standard for plasma protein, time 1 biopsy, and time 2 biopsy were -28.424 ± 0.3, -27.416 ± 0.4, and -26.626 ± 0.8 (δ^13^C_PDB_), respectively. The δ^13^C_PDB _difference between sequential biopsies was 0.789 ± 0.1. The δ^13^C_PDB _difference between plasma protein enrichment and time 1 biopsy was 1.009 ± 0.3. The δ^13^C_PDB _difference between plasma protein enrichment and biopsy at time 2 was 1.798 ± 0.6.

#### Myofibrillar protein synthesis

**Figure 2:**
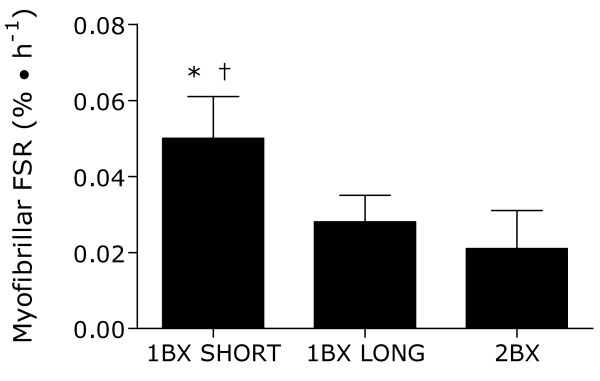
**Myofibrillar protein fractional synthetic rate (FSR) calculated by**. 1) utilizing plasma protein enrichment and a short incorporation time (90 min; 1BX SHORT), 2) utilizing plasma protein and a long incorporation time (240 min; 1BX LONG), or 3) utilizing sequential muscle biopsies (2BX) in study 1. *Significantly different from 2BX, P < 0.001. †Significantly different from 1BX LONG, P = 0.002. Values are means ± SD; n = 8

**Figure 3:**
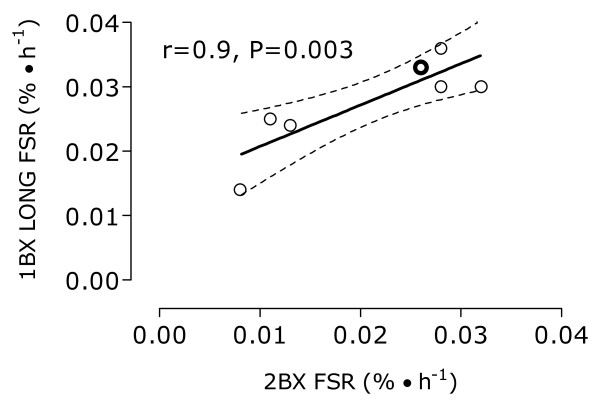
**The relationship between obtaining sequential muscle biopsies (2BX) and utilizing the single biopsy approach with a long incorporation time (1BX LONG) in study 1**. There was a significant (r = 0.9, P = 0.003) correlation between rates of myofibrillar protein synthesis (FSR, %/hr) using the 2BX and 1BX LONG approaches. Dark line: line of linear best fit, with 95% confidence interval, for 1BX LONG versus 2 BX - y = 0.64 ± 0.16x + 0.01 ± 0.001. Dashed line: line of identity. Note: only 7 points are visible since two points share the same values (outline in bold).

Myofibrillar protein synthesis rates were not different (Figure 2; P = 0.3) between 1BX LONG and 2BX and were correlated (Figure 3; r = 0.9, P = 0.003). The intercept for the linear regression of best fit between 1BX LONG with 2BX was 0.01 ± 0.001 and the slope of this regression was 0.6 ± 0.1. The percent and absolute changes in rates of myofibrillar protein synthesis between 1BX LONG and 2BX were 23 ± 8% and 0.007 ± 0.005%/hr, respectively. However, 1BX SHORT was significantly different from 1BX LONG (P = 0.002) and 2BX (P < 0.001). There was no relationship between 1BX SHORT and 2BX (r = -0.2, P = 0.6). The percent and absolute differences in rates of myofibrillar protein synthesis between 1BX SHORT and 2BX were 61 ± 21% and 0.024 ± 0.012%/hr, respectively.

### Study 2: Steady-state confirmation

#### Plasma and muscle intracellular free phenylalanine enrichment

**Figure 4:**
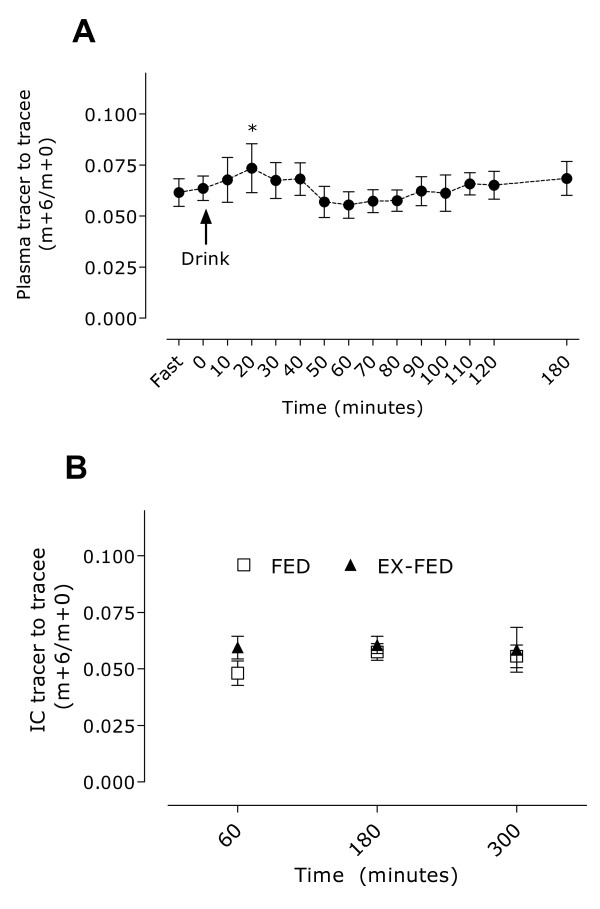
**Plasma (A) and muscle intracellular free (B) phenylalanine enrichments (tracer·Tracee^-1^) after resistance exercise and protein ingestion (EX-FED) or protein ingestion a lone (FED) in study 2**. *Significantly different from pre value, P < 0.001. All values are means ± SD; n = 8.

Plasma free and muscle intracellular free phenylalanine enrichments are shown in Figure 4, A and 4B, respectively. Plasma free enrichments were significantly different (P < 0.001) from pre-drink ingestion at 20 min after drink ingestion. However, linear regression analysis indicated that the slopes of the plasma free enrichments over time were not significantly different from zero (P = 0.8), suggesting that plasma free enrichments had reached a plateau and subjects were at isotopic steady-state over the duration of the infusion. Moreover, the slope of the intracellular phenylalanine enrichment (i.e., the precursor pool used in all of our previous studies) by time curve for both FED and EX-FED was not different from zero (P = 0.4 and 0.7, respectively), giving further confirmation that steady-state conditions were achieved. Coefficients of variation in tracer enrichment after drink consumption in the plasma free (i.e. from fast to 180 min; Figure 4A) and IC pools (i.e. from 60 - 300 min; Figure 4B) were 12 ± 2% and 6 ± 2%, respectively.

#### Blood amino acid concentrations

**Figure 5:**
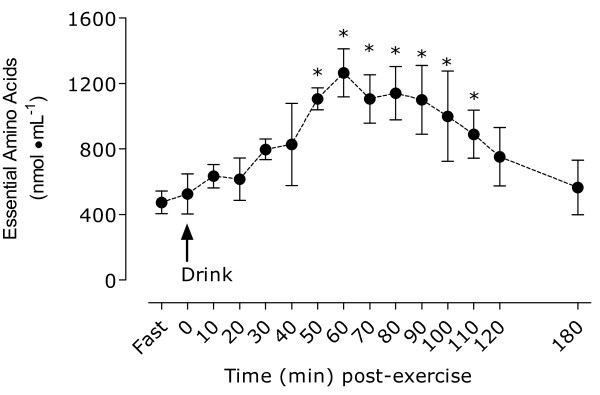
**Blood concentrations of essential amino acids after ingestion of 25 g of whey protein isolate in study 2**. *Significantly different from pre value, P < 0.05. All values are means ± SD; n = 8.

Essential amino acid (EAA) concentrations are shown in Figure 5. Protein ingestion induced a significant rise in EAA concentration at 50 min and this elevation was maintained until 110 min (all, P < 0.05).

## Discussion

The present study demonstrates that using proteins isolated from plasma for baseline enrichments, coupled with a carbon-based tracer and analysis of protein from a single muscle biopsy by GC-C-IRMS, serves as a reliable alternative to obtaining two biopsies for the calculation of myofibrillar protein synthesis. However, the length of this initial incorporation period needs to be considered with the one biopsy approach. As a rule, the reliability of the single biopsy approach is dependent on the use of subjects who have never received tracer before participation in the study (i.e. "tracer naїve") as the muscle, due to its slow turnover, will have residual tracer enrichments remaining from previous infusion experiments. To the authors' knowledge it remains to be systematically investigated the length of time in which ^13^C muscle enrichments return to pre-infusion values; however, such an effect could take years and contain substantial between-subject variations based on age or activity level. Thus, we do not know the time required to reinstate a participant into a tracer infusion protocol that employed the single biopsy approach.

It is clear that assuming zero background enrichment to calculate rates of muscle protein synthesis is an egregious approach [3] and should not be confused with the technique employed in the current study (i.e., plasma protein for baseline enrichment), which we have used previously [4-6]. The finding that a short incorporation period (i.e., 1BX SHORT) results in inflated rates of myofibrillar protein synthesis arises from the fact that a 1.009 δ^13^C_PDB _change occurred from the plasma protein ^13^C enrichments during a short time frame (i.e., 88 min). This finding is not entirely surprising as there can be considerable variation, in our hands, between subjects in the change in muscle enrichments (i.e. δ^13^C_PDB_) when attempting to calculate FSR during 0 - 1 h time intervals. Therefore, it is likely an artifact of the approach and it is possible that this arises from the non-steady state conditions that existed in the initial period after initiating the infusion. However, this effect becomes trivial over a larger time interval (3 h) of incorporation (i.e., 1BX LONG). It should be acknowledged that protein-bound tracer enrichments can be derived by using GC-MS; however, when utilizing a deuterated tracer (e.g.[^2^H_5_]phenylalanine), the amino acid derivative is especially vulnerable to isotope effects during fragmentation inside the ionization source during analysis [27]. These non-equating ion abundance ratios can be corrected by a standard curve to better represent the true tracer-to-tracee ratio [27], but this would not be possible in a baseline sample in which no tracer (i.e., a detectable m+5 or an m+6 ion using GC-MS for analysis) is present. Therefore, the current study is specific to carbon-based tracers and utilizing GC-C-IRMS to determine the enrichments of the labelled amino acid in the plasma protein bound sample.

It is important to highlight that the 1BX LONG condition did result in slightly elevated myofibrillar protein synthesis rates, although not statistical significant, from that of the 2BX that may arithmetically influence the relative change in FSR observed after a feeding and/or an exercise stimulus (i.e. the observed difference may be slightly less than that observed after the 2BX condition). Regardless, we have detected robust changes in muscle protein FSR after feeding 15 g of whey protein at rest [23], resistance exercise in the fasted-state [5], and after a combined feeding and exercise stimulus [4,6], which further demonstrates that it is possible to detect changes in FSR with the 1BX LONG approach. However, under certain circumstances, where large increases in muscle protein FSR may not be expected (e.g. feeding a minimal amount of protein to elderly subjects), the 2BX approach may be worth considering. A final point of concern was the 1BX LONG produced a muscle protein FSR that was 23% greater than that observed in the 2BX condition. However, a sample size of 47 participants would be necessary to manifest statistically significant differences between conditions based on power calculations performed using a two sided statistical test, a false positive rate of 5%, and a power of 80%.

A second objective of the study was to provide a direct validate to an approach that we have used to determine rates of muscle protein synthesis after bolus protein ingestion to determine protein feeding- and/or exercise plus protein feeding-induced changes in muscle protein synthesis [4,6,18]. Specifically, we wished to present detailed evidence confirming that adding a small amount of labelled free amino acids (i.e., tracer) to a protein drink does not disrupt steady-state conditions in sampled precursor pools. Here, we established that steady-state conditions were not substantially disrupted in the plasma free or, most importantly, the IC protein pools over the duration of the infusion, as noted by the slope of the plasma free and IC enrichments by time curve were not significantly different from zero during the infusion. Since an appropriate level of tracer was added to the protein to prevent 'over-enriching' the drink we did not create a large rise in enrichment to disturb steady-state conditions, which has been reported by others [20]. As expected, the coefficients of variation in the sampled precursor pools for plasma (12%) and IC free amino acid pools (6%) did illustrate that the plasma free pool is more 'vulnerable' to changes in enrichment from tracer enriched protein drinks as compared to the IC pool. It is worth mentioning, however, that we agree with previous suggestions that it is not advisable to assess digestion and absorption kinetics by adding tracer to a protein drink and therefore, intrinsically labelled dietary proteins are of tremendous value in this regard [28].

## Conclusion

In conclusion, we supply direct evidence demonstrating that a single biopsy approach, in tracer naїve participants, serves as a reliable technique compared to obtaining an additional biopsy to determine muscle protein FSR. This finding has practical significance as it will minimize the discomfort and associated risk associated with multiple biopsies. However, based on our data the tracer incorporation should be long enough (i.e., ~3 hours from our results) to reliably determine rates of myofibrillar protein synthesis. In addition, adding appropriate quantities of crystalline tracer to a bolus protein drink does not disrupt steady-state conditions and in fact aids in minimizing perturbations in the most valid precursor pool (i.e., the IC pool), thus providing appropriate conditions for the application of the steady-state precursor product equation.

## Competing interests

The authors declare that they have no competing interests.

## Authors' contributions

SMP acquired the research grant. NAB, DWDW, and SMP participated in the design of the study. NAB, DWDW, TR, TP, SKB, and SMP carried out the study. NAB, DWDW, TR, and TP conducted laboratory analyses. NAB and SMP performed statistical analyses and wrote the manuscript. All authors read and approved the final manuscript.
